# Human Herpesvirus-8 (HHV-8)-Positive Human Immunodeficiency Virus (HIV)-Negative Multicentric Castleman Disease With a Fulminant Course

**DOI:** 10.7759/cureus.54350

**Published:** 2024-02-17

**Authors:** Maria Teresa Brito, Ana Filipa Amador, Ricardo Moço Coutinho, Ana Ribeiro, Jorge S Almeida

**Affiliations:** 1 Internal Medicine, Centro Hospitalar Universitário de São João, Porto, PRT; 2 Cardiology, Centro Hospitalar Universitário de São João, Porto, PRT; 3 Allergy and Immunology, Centro Hospitalar Universitário de São João, Porto, PRT; 4 Medicine, Faculdade de Medicina da Universidade do Porto (FMUP), Porto, PRT

**Keywords:** human herpesvirus-8, plasmablastic variant, pancytopenia, lymphoproliferative disorder, multicentric castleman disease

## Abstract

Multicentric Castleman disease (MCD) is a poorly understood, heterogeneous lymphoproliferative disorder with benign hyperplastic lymph nodes and systemic inflammatory symptoms. Human herpesvirus-8 (HHV-8) may be associated with MCD, whether or not the patient is infected with the human immunodeficiency virus (HIV). A 74-year-old man presented with anaemia, thrombocytopenia and bilateral axillary adenomegaly of unknown origin. The patient was admitted to the hospital two years ago with clinical signs of weight loss, asthenia, anorexia and a maculopapular rash on the trunk and back. Blood analysis showed pancytopenia (haemoglobin 7.7 g/dL, leucocytes 2.55 x 10^9^/L and platelets 41 x 10^9^/L), elevated acute phase reactants (such as C-reactive protein, erythrocyte sedimentation rate, ferritin and fibrinogen), hypoalbuminemia and hypergammaglobulinemia, and HIV serology was negative. Thoracic, abdominal and pelvic axial tomography showed generalised lymphadenopathy. The bone marrow biopsy showed only reactive changes, and the histology of an excisional biopsy of the adenopathy was consistent with the plasmablastic variant of MCD associated with HHV-8. The HHV-8 viral load was 3.8 x 10^4^ copies/mL (4.5 log). He was started on prednisolone 60 mg/day and rituximab. He had a poor response to therapy, despite a reduction in the HHV-8 viral load, with clinical deterioration, transfusion-dependent anaemia and progression to multi-organ dysfunction leading to death three weeks after starting treatment. Our patient had a fulminant course of MCD despite treatment with rituximab. Further studies are needed to validate the different treatment modalities and to better understand the prognosis of this disease.

## Introduction

Castleman disease (CD) is a heterogeneous group of lymphoproliferative disorders of unknown aetiology, also known as angiofollicular lymph node hyperplasia [1]. Some CDs share common histopathological features, but, due to different clinical characteristics, they have different clinical complications, treatment and outcomes. It is therefore divided into two types: a localised form involving one or more enlarged lymph nodes in a single region of the body and usually asymptomatic (unicentric disease) and a multicentric form characterised by fever with chills, anaemia, generalised lymphadenopathy, hepatosplenomegaly and a more aggressive behaviour [2]. In addition, CD has different histological patterns: the hyaline vascular, plasma cell and plasmablastic (a subvariant of the plasma cell variant), the latter two of which are more commonly associated with multicentric Castleman disease (MCD) [3,4].

MCD is also subclassified according to the presence of human herpesvirus-8 (HHV-8), which is found in approximately 13% of patients. HHV-8 is currently known to be present in 100% of MCD cases in human immunodeficiency virus (HIV)-infected patients and in 40-50% of HIV-negative cases [5]. It is estimated that there are 6,500-7,700 new cases of CD each year in the USA, of which 25% are multicentric. The median age at diagnosis is 50-65 years and 50-65% are male. MCD has a more aggressive natural history in HIV-negative patients and typically presents in the sixth decade of life [5]. 

The pathogenesis of MCD is not understood, and its treatment remains uncertain due to the low incidence of CD. The clinical, laboratory and treatment outcomes of CD are mainly based on case reports and small series studies [6]. Data on HHV-8-associated CD in HIV-negative patients is even more scarce [4]. Here, we report a case of an HIV-seronegative patient with HHV-8 positive CD and describe the clinical, pathological and laboratory features of the disease.

## Case presentation

A 74-year-old man of Mediterranean descent was admitted for pancytopenia (haemoglobin 7.7 g/dL, leukocytes 2,550 x 10^9^/L and platelets 41,000 x 10^9^/L) with constitutional syndrome - fever, weight loss, asthenia, anorexia, jaundice and pruritic maculopapular rash on the trunk and back - progressing over the last two months. There were no lesions suggestive of Kaposi's sarcoma.

Two years earlier, he had been followed up in the haematology department for stable and asymptomatic bicytopenia (haemoglobin 7.3 g/dL, platelets 40 000 x 10^9^/L) and bilateral axillary adenomegaly (the largest measuring 25 x 14 mm), with inconclusive investigations (including excision of the adenomegaly and bone marrow biopsy) and laboratory improvement after a short cycle of corticotherapy.

The present blood test results showed elevated acute phase reactants, including erythrocyte sedimentation rate 140 mm/1ªh, C-reactive protein 111.3 mg/L, ferritin 1388 ng/mL and fibrinogen 522 mg/dL, with negative serological markers, including for HIV and all studies of autoimmune disorders. The patient had no complement consumption. A peripheral blood smear showed only anisopoikilocytosis without schizocytes or spherocytes. Serum protein electrophoresis showed a gamma peak, and peripheral blood immunophenotyping showed changes compatible with incomplete lambda monoclonal gammopathy.

**Figure 1:**
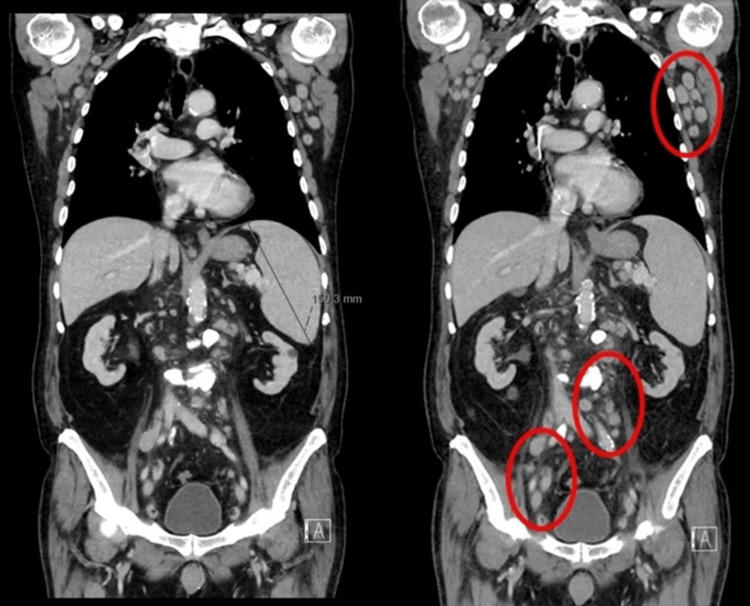
Chest and abdominal computed tomography Adenomegaly (bilateral axillary adenomegaly with slight growth compared to the previous examination, the larger on the left at 25 x 16 mm), small pleural effusion and splenomegaly (15 cm bipolar diameter).

A skeletal X-ray was performed to exclude multiple myeloma without areas suggestive of lytic bone involvement. Chest and abdominal computed tomography showed thoraco-abdomino-pelvic adenomegalies (bilateral axillary adenomegaly with a slight growth compared to the previous examination, the larger on the left with 25 x 16 mm), small pleural effusion and splenomegaly (15 cm bipolar diameter) (Figure 1).

**Figure 2:**
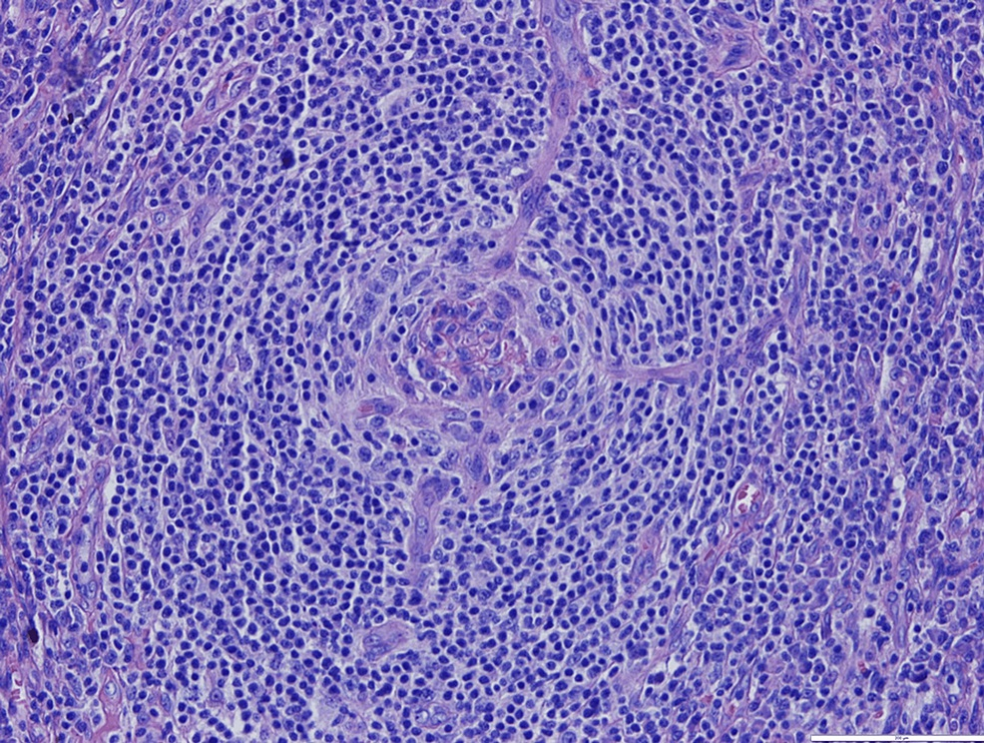
Germinal center depleted with an onion skin arrangement of lymphocytes and some plasmablasts

**Figure 3:**
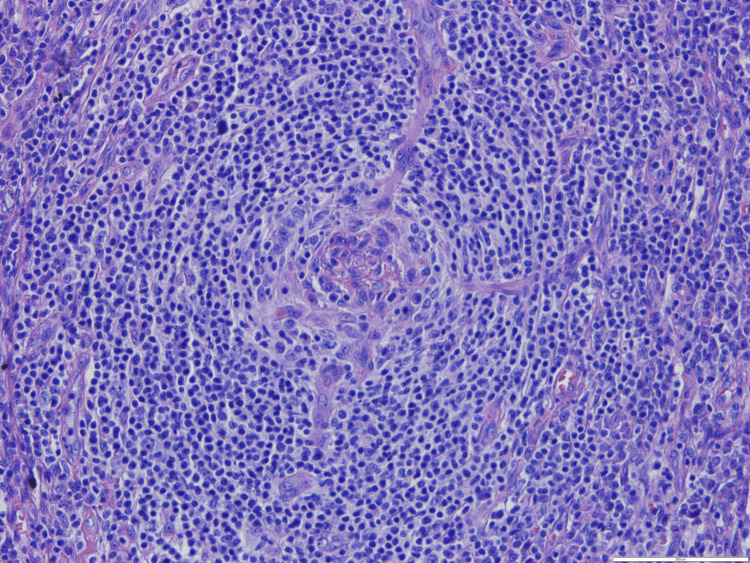
Germinal center in lollipop

**Figure 4:**
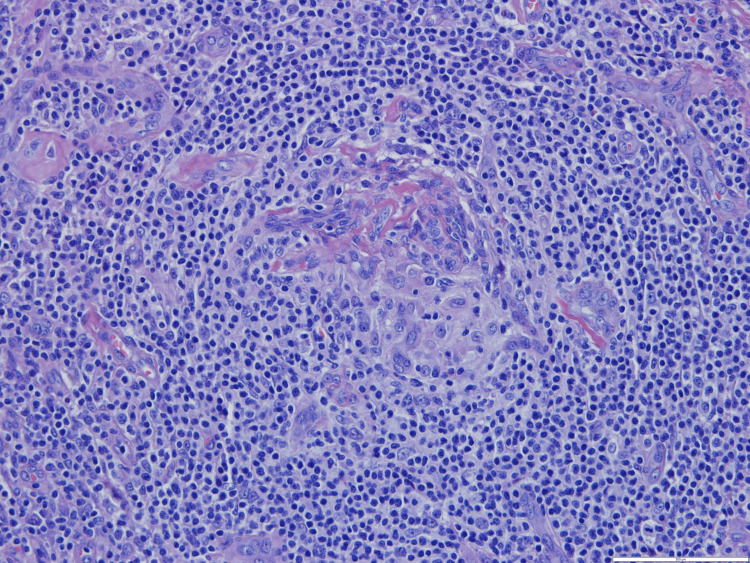
Great magnification of the germinal center

**Figure 5:**
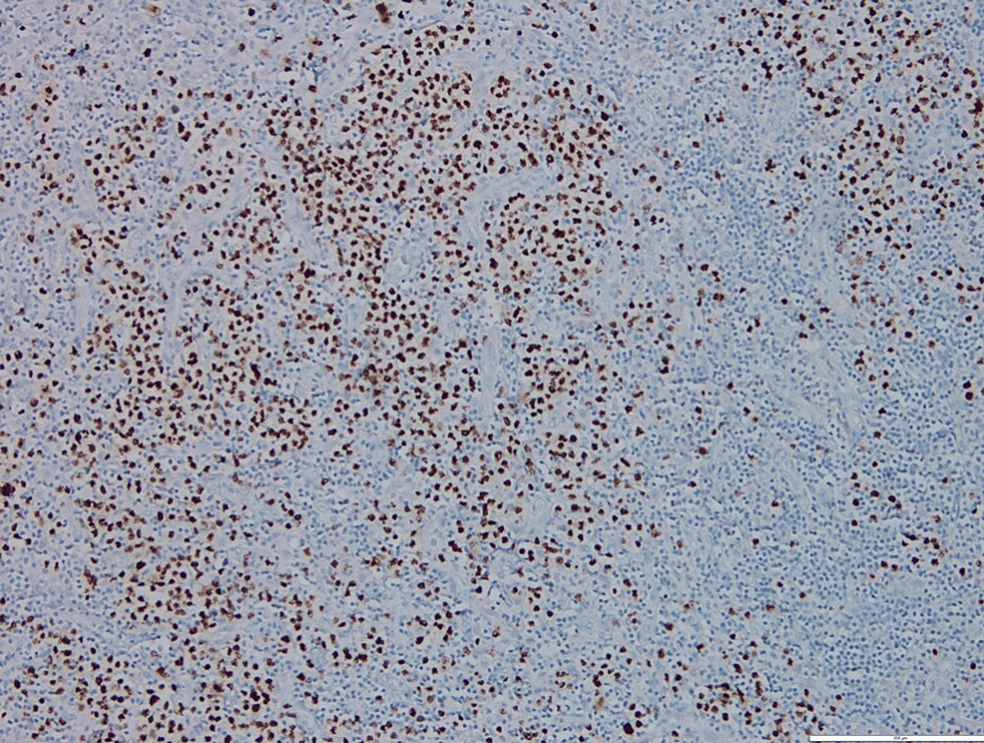
Interfolicular HHV-8

A bone marrow biopsy was performed, which showed only reactive and myelodysplastic changes, but an excisional biopsy of the adenopathy showed an abundant population of plasma cells with plasmablasts in the perifollicular location with a positive HHV-8 test (Figures 2, 3, 4, 5). The blood HHV-8 viral load was 3.8 x 104 copies/mL (4.5 log).

He was started on steroid therapy (prednisolone 60 mg/day) and weekly rituximab therapy (635 mg ev calculated on the basis of a dose of 375 mg/m^2^ of the body surface area). Doxorubicin was administered pending ethical approval. Despite a decrease in the HHV-8 viral load (3.8 x 104 copies/mL (4.5 log)), clinical and laboratory deterioration occurred, with worsening clinical condition, transfusion-dependent anaemia and thrombocytopenia with minor blood loss. Progression to multiorgan dysfunction and death occurred 34 days after the start of the treatment.

## Discussion

There is no standard treatment, as only a small number of case reports or small groups of cases have been described in the different studies. It is difficult to recommend a specific treatment for MCD, and available treatments include corticosteroids, mono- and polychemotherapy, antivirals, interferon-alpha, IL-6 antibodies, IL-6 receptor antibodies and CD20 monoclonal antibodies [1,6].

The aetiology of MCD is not fully understood, but HHV-8 appears to play a role, particularly in HIV-positive cases [3]. HHV-8 infects immunoglobulin M light chain-restricted plasmablasts in the mantle zone, and replicating HHV-8 encodes a viral IL-6. The dysregulated overproduction of IL-6 by lymph nodes can cause the classic clinical picture [7].

The variable expression of CD20, a phosphoprotein that is expressed on the surface of B lymphocytes, is a characteristic feature of infected lymphoid cells. Rituximab is an anti-CD20 antibody and could therefore be a possible therapeutic target in MCD by eliminating lymphocytes infected with HHV-8 human herpes virus 8 [3]. In prospective studies of HIV-positive MCD, rituximab improved constitutional symptoms and produced objective responses in up to 70% of patients [8]. It was also shown to improve overall survival. As a result, rituximab has become the standard first-line treatment for HIV-positive MCD. Data are scarce in HIV-negative disease. Few reports have described remissions after rituximab [9]. The lack of symptomatic and analytical improvement with rituximab therapy in our case contradicts what is described in the literature. It would be interesting to investigate whether advanced age or exuberance of symptoms at diagnosis alters the likelihood of response to rituximab.

Particularly, in symptomatic and advanced diseases, such as the case of our patient, there is no therapeutic consensus for MCD, and combination regimens with chemotherapies, such as cyclophosphamide or doxorubicin, are also available as treatment options [3]. With few exceptions, the various chemotherapy regimens that have been evaluated in HHV-8-positive, HIV-negative patients have resulted in poor outcomes [9].

On the other hand, tocilizumab is an anti-IL-6 receptor antibody that blocks the IL-6-induced inflammatory pathway. It is worth investigating whether tocilizumab is effective in HHV-8-associated MCD cases because vital IL-6 does not require the subunit to subsequently activate the JAK-STAT pathway [10].

With regard to the HHV-8 load, the relationship between the HHV-8 viral load in HIV-negative patients and these diseases, particularly MCD, remains unclear. There are some data suggesting that reduced HHV-8 viral load correlates with responding to the treatment. Therefore, HHV-8 viremia may be a common finding in suspected MCD relapse [9]. In our patient, despite the decrease in the HHV-8 viral load, the outcome was poor.

Broad-spectrum antivirals targeting HHV-8 are not part of the routine management of MCD or Kaposi's sarcoma because they have not been shown to be effective. This may suggest that MCD, once clinically manifested, is independent of viral control [7,9].

MCD has a slowly progressive course with a poor outcome, but the natural history is variable. It is difficult to assess prognosis due to a lack of understanding of clinical features and outcome factors. The five-year overall survival rate has been estimated at 42% for patients with HHV-8-positive MCD and 75% for those with HHV-8-negative MCD [1]. Our patient had some characteristics that would indicate a poor prognosis, such as advanced age at diagnosis, multicentric type, HHV-8 positivity and plasmablastic variant. Nevertheless, such a fulminant course has rarely been described in the literature.

Finally, in our case, similar to other cases described in the literature, there was no evidence of immunodepression. Some authors have suggested an age-related specific immunodeficiency leading to loss of HHV-8 control [4].

## Conclusions

Sixty years after the initial description of a new clinicopathological entity by Benjamin Castleman, the spectrum of CD has broadened considerably and now includes several distinct diseases. Because of the uncertain prognosis of MCD, a careful diagnostic workup to identify and rule out all differential diagnoses is essential to achieve the correct diagnosis for appropriate therapy. Although a number of therapeutic interventions are used to treat these patients, none offer a cure, and there are no clinical guidelines, standardised clinical criteria or clinical consensus statements. Clearly, studies with larger populations and longer follow-ups are needed to confirm the effectiveness of different treatments.
